# FAST-EM array tomography: a workflow for multibeam volume electron microscopy

**DOI:** 10.1515/mim-2024-0005

**Published:** 2024-07-11

**Authors:** Arent J. Kievits, B. H. Peter Duinkerken, Ryan Lane, Cecilia de Heus, Daan van Beijeren Bergen en Henegouwen, Tibbe Höppener, Anouk H. G. Wolters, Nalan Liv, Ben N. G. Giepmans, Jacob P. Hoogenboom

**Affiliations:** Department of Imaging Physics, Delft University of Technology, Delft, The Netherlands; Department of Biomedical Sciences, University Medical Center Groningen, Groningen, The Netherlands; Center for Molecular Medicine, University Medical Center Utrecht, Utrecht, The Netherlands

**Keywords:** volume electron microscopy, FAST-EM, array tomography, image processing, automatic segmentation

## Abstract

Elucidating the 3D nanoscale structure of tissues and cells is essential for understanding the complexity of biological processes. Electron microscopy (EM) offers the resolution needed for reliable interpretation, but the limited throughput of electron microscopes has hindered its ability to effectively image large volumes. We report a workflow for volume EM with FAST-EM, a novel multibeam scanning transmission electron microscope that speeds up acquisition by scanning the sample in parallel with 64 electron beams. FAST-EM makes use of optical detection to separate the signals of the individual beams. The acquisition and 3D reconstruction of ultrastructural data from multiple biological samples is demonstrated. The results show that the workflow is capable of producing large reconstructed volumes with high resolution and contrast to address biological research questions within feasible acquisition time frames.

## List of abbreviations

EM: electron microscopy
vEM: volume electron microscopy
SEM: scanning electron microscopy
TEM: transmission electron microscopy
bd-TEM: beam-deflection transmission electron microscopy
ATUM: automated tape-collecting ultramicrotomy
mSEM: multibeam scanning electron microscopy
STEM: scanning transmission electron microscopy
OSTEM: optical scanning transmission electron microscopy
FAST-EM: fast automated scanning transmission electron microscopy
MPPC: multipixel photon counter
ROA: region of acquisition

## 1 Introduction

Unraveling the complexities of biology across various scales, from organs down to cells and biomolecules needs a full understanding of biological (ultra)structure. Traditionally, electron microscopy (EM) has been used to decipher tissue and cellular ultrastructure, using mainly 2D micrographs of selected areas. However, conventional EM fails to provide the context needed for reliable biological interpretation. In recent years, EM techniques collectively known as large-scale and volume electron microscopy (vEM) have emerged, offering unprecedented insights into the 3D structures of biological specimens at the nanoscale [1]. While these techniques have proven their value, the limited sustained throughput has hindered their ability to handle large volumes of samples effectively, thus restricting the scope of vEM [2].

To address the throughput limitations of electron microscopes, multiple approaches have been developed. Traditionally, vEM techniques can be divided into scanning EM (SEM) and transmission EM (TEM) techniques. vEM with TEM is based on imaging of serial ultrathin sections. The main throughput-limiting factors are the field-of-view (FOV) as set by the detector, slow stage movements and limited observable sample area as determined by the sample grids. The limited FOV of the camera and stage movements have been addressed by TEM camera array (TEMCA, [3], [4], [5]). The FOV that can be imaged with a single stage movement has further increased with beam-deflection TEM (bd-TEM, [6]). Multiple systems can be used in parallel to further boosts acquisition speeds [7]. Additionally, an electron-transparent tape-based reel system (GridTape) can be used that significantly reduces the number of necessary vacuum cycles [8]. These developments have addressed the most important throughput limiting factors of TEM.

The main focus in vEM techniques that utilize SEM has been to improve the scanning speed, which is limited by the maximum probe current allowing for high resolution imaging. Approaches have been developed that circumvent the probe current limitation using multiple parallel scanning beams, effectively boosting the scanning speed by orders of magnitude. A few implementations of multibeam SEM (mSEM) exist, including MultiSEM based on secondary electron imaging [9] and FAST-EM based on transmission imaging [10], [11].

The throughput increase achieved by TEMCA, bd-TEM and mSEM, in combination with an approach for generating large amounts of sections such as automated tape-collecting ultramicrotomy (ATUM, [12]), has made it possible to image millimeter-sized samples [6], [7], [13], [14]. However, these techniques have thus far been accessible only to a limited number of research groups and used in a narrow scope of applications. While mSEMs and GridTape have recently become commercially available, additional requirements may create new challenges for sample preparation and possible applications. The reliance of bd-TEM on electron-transparent tape complicates the section collection and handling with added risks of support film breakage and off-slot collection. In MultiSEM, the combination of a high applied bias voltage and secondary electron detection may impose restrictions on sample staining, conductivity and height tolerances.

**Figure 1:**
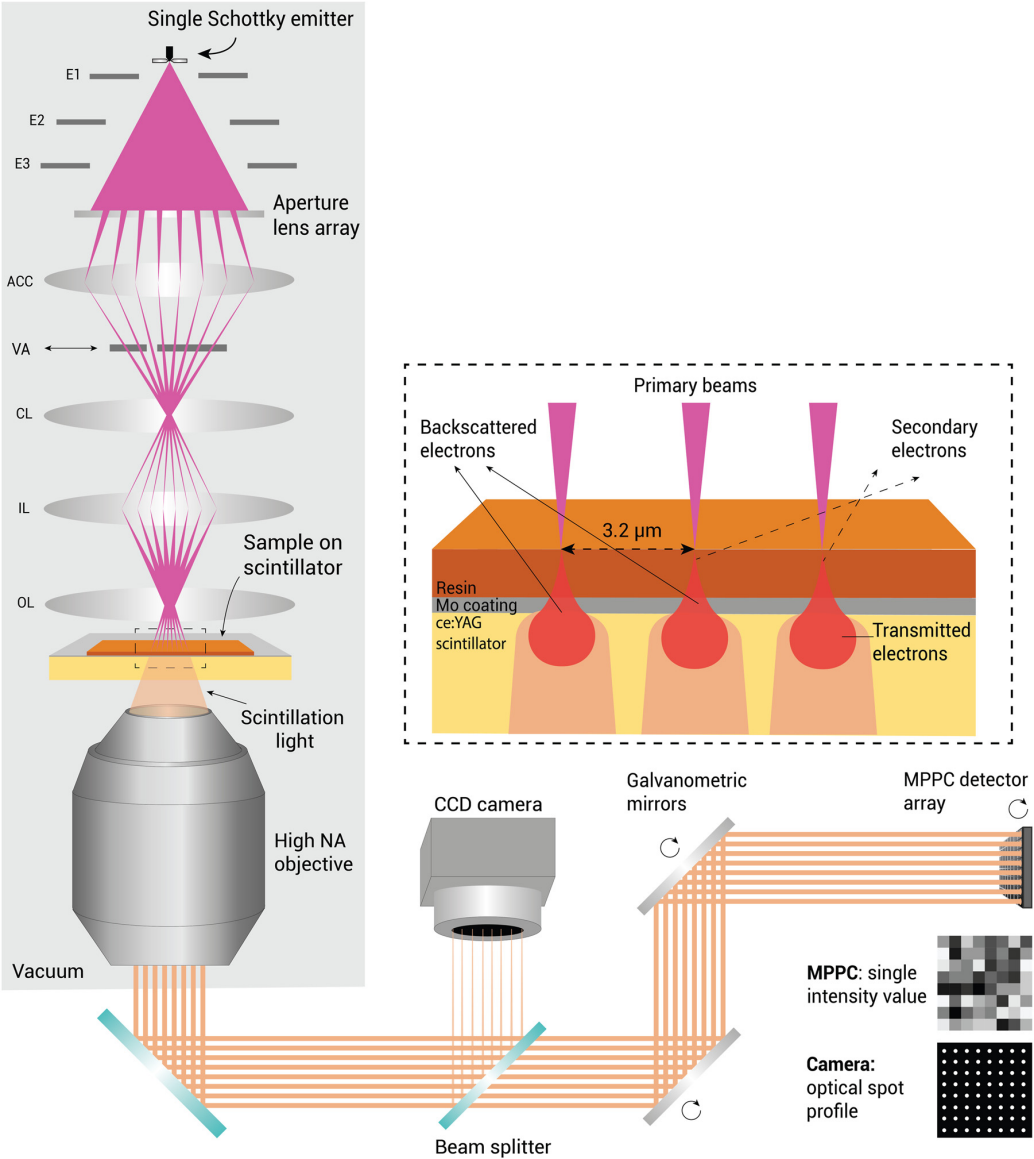
FAST-EM principle. An aperture lens array splits the emission cone of a single high brightness Schottky source into an array of 8 by 8 electron beams (implementation is described in [17]). The beams scan the sample in parallel with a 3.2 µm pitch. A single beam can be selected with a variable aperture (VA). The transmitted electrons are converted into photons by a scintillator substrate and collected by a high NA objective lens. An optical system outside of the vacuum chamber (shown simplified) then descans and magnifies the optical spots and projects them onto a multipixel photon counter (MPPC) array. A CCD camera situated outside the main optical path monitors the spot profile. E1/E2/E3: Source electrodes; ACC, accelerator lens; VA, variable aperture; CL, condenser lens; IL, intermediate lens; OL, objective lens.

We demonstrate mSEM imaging with the FAST-EM (Fast, Automated Scanning Transmission Electron Microscopy). FAST-EM uses a recently introduced optical transmission detection technique to separate the electron beam signals, referred to as optical STEM or OSTEM [15], [16]. In OSTEM, ultrathin sections are mounted on a scintillator that converts electrons transmitted through the section into photons, which are then collected by an objective lens below the scintillator and guided to a detector array (Figure 1). This signal generation and detection principle was recently characterized and shown comparable to commonly used backscattered electron detection in terms of contrast, resolution, and signal-to-noise ratio (SNR) [15]. Here, we report an array tomography workflow for vEM with an early adopter FAST-EM system. We performed acquisitions on cultured cells as well as tissue samples and demonstrated the wide applicability of FAST-EM. As an example, FAST-EM array tomography was applied to cultured cells, reconstructing a 265,000 μm^3^ volume from 72 thin serial sections with 4 × 4 × 100 nm^3^ voxel size and resolving the mitochondrial cristae and membrane structures. Our results show that FAST-EM is capable of imaging large unobstructed regions of interest with feasible acquisition times, while providing images with high resolution and contrast to address biological research questions.

## 2 Results

### 2.1 FAST-EM array tomography

**Figure 2:**
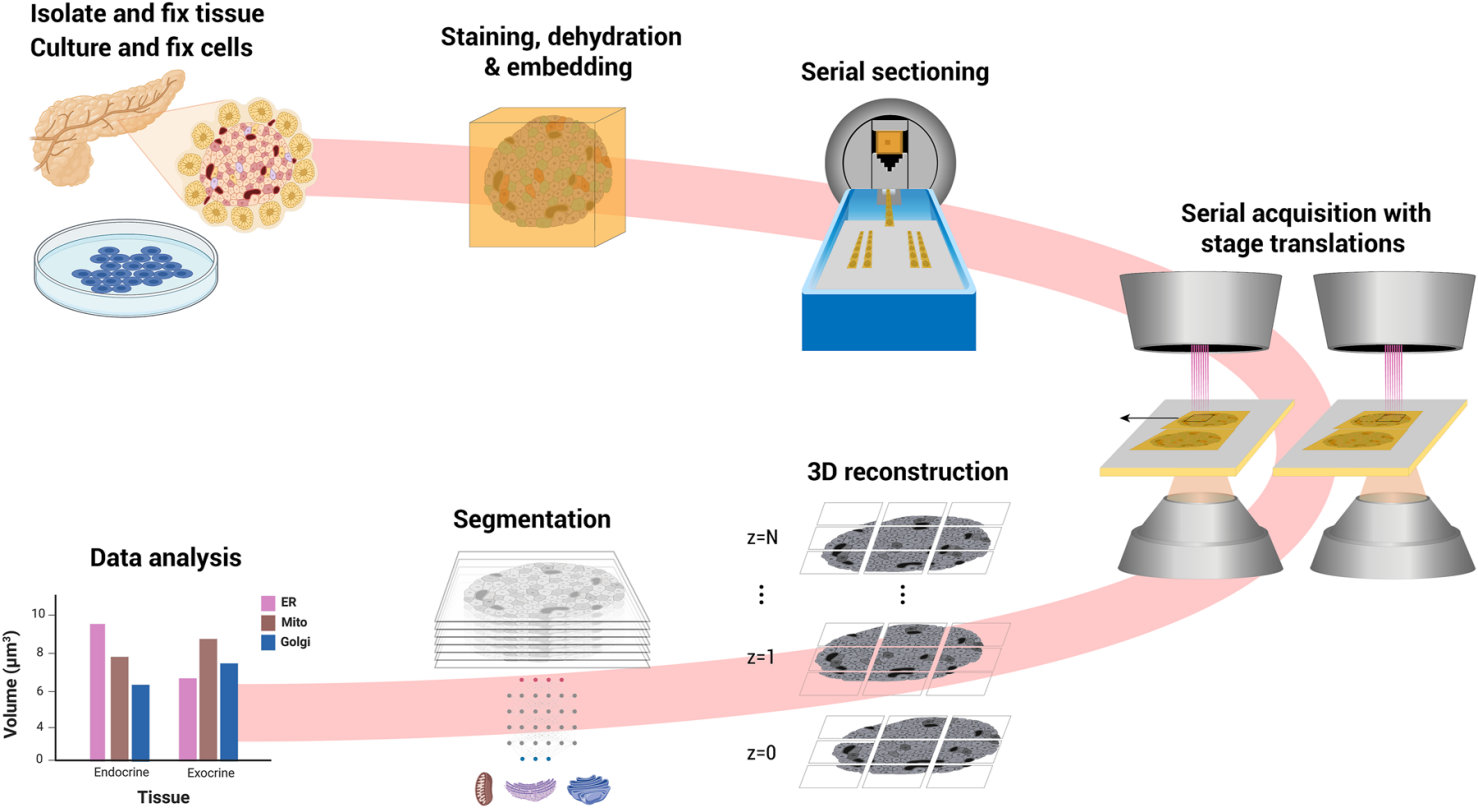
FAST-EM array tomography. Tissue or cultured cells are fixed, contrasted with heavy metals, dehydrated and embedded in epoxy resin. Ultrathin sections are deposited on a molybdenum-coated, cerium-doped yttrium aluminum garnet (ce:YAG) scintillator crystal in the knife bath. The sections are imaged using mosaicking with stage increments. The 3D volume is reconstructed from 2D images. Features of interest are (semi-)automatically segmented. Data analysis is performed on the segmentation results. Figure partially created with BioRender.com.

In FAST-EM array tomography, serial sections are cut from resin-embedded tissue or cells and collected onto scintillator substrates (Figure 2), similar to conventional approaches [18], [19]. Serial sections are imaged sequentially, incrementing the stage and sample at fixed intervals to acquire areas larger than the multibeam field-of-view with overlap between individual images. A continuous volume is reconstructed from the 2D images using point correspondences sought in the overlap region between images in 2D and 3D. The aligned volume can then be segmented and analysed.

FAST-EM employs a light optical system to collect, descan, and detect scintillation photons that are produced when the electron beams scan the sample (Figure 1). The electron beams are arranged in an 8 by 8 square pattern (also referred to as *multiprobe*), created by an aperature array in the electron source module. They scan at a pitch of 3.2 µm to ensure sufficient separation on the detector array of the optical spots produced by each beamlet. The photons generated in the scintillator from the 64 beamlets are collected by a high NA in-air objective lens situated directly under the sample holder in the vacuum chamber, and projected onto a set of galvonometric mirrors that perform a descanning in both *x* and *y* directions. Approximately 5 % of the photon intensity is split to a CCD camera outside of the main optical path to monitor the optical spot profile during acquisition. The remaining photons are directed onto a multipixel photon counter (MPPC) array which produces a single intensity readout for each beamlet at each scan position, building up the transmission electron image. The optical system ensures rapid electron detection and stable image quality over a prolonged acquisition time.

### 2.2 FAST-EM image acquisition

**Figure 3:**
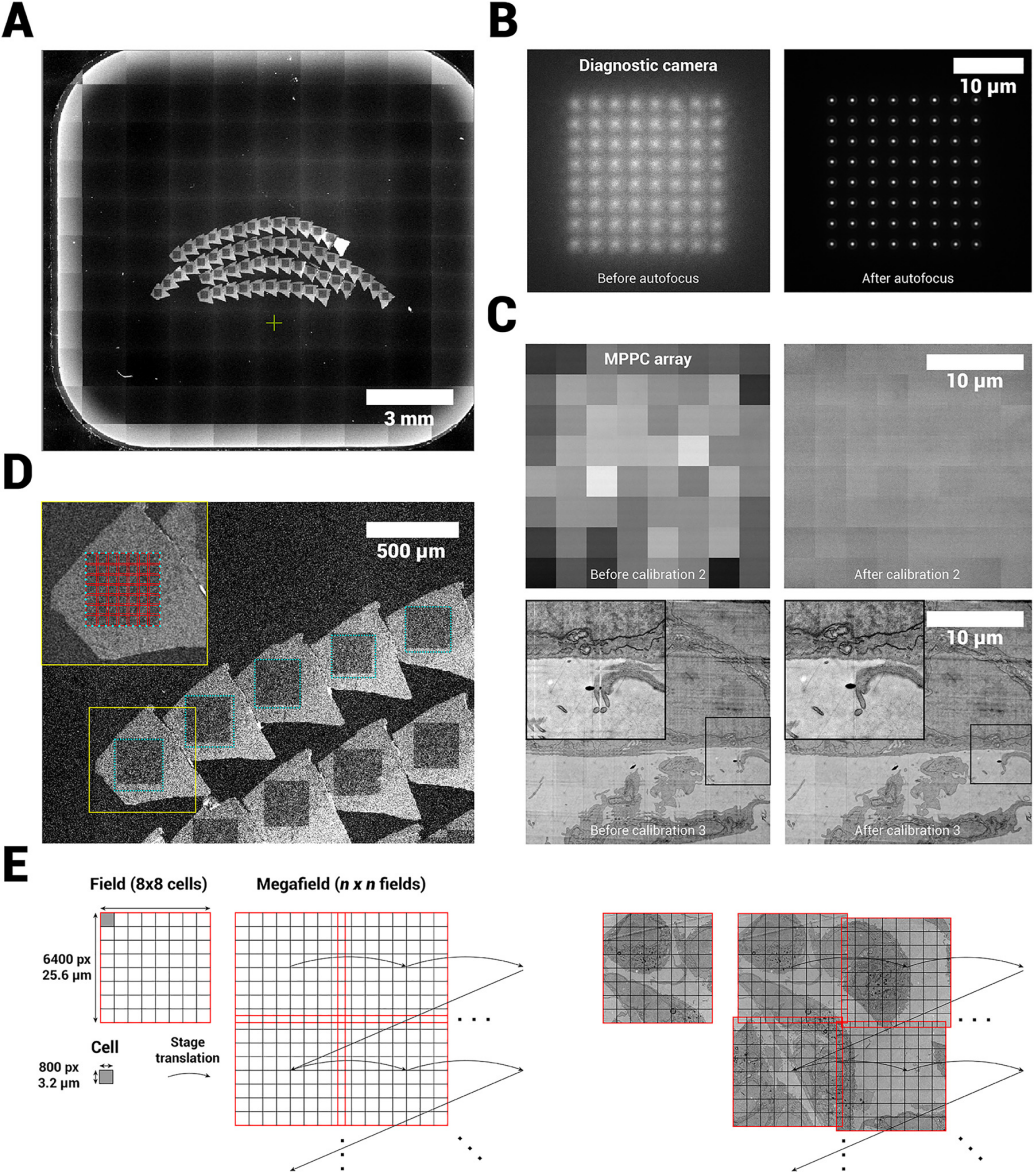
Acquisition workflow for FAST-EM. A Overview images are acquired to guide ROA definition and calibration region selection. B Diagnostic camera images of spot profile before and after optical focus calibration. C Single field image (MPPC detector) before and after digital offset and gain calibration (upper row) and before and after cell translation calibration (lower row). D Zoom in on overview image showing the ROAs on the sample, and the approximate division of a ROA into fields. E Terminology and acquisition order of a single ROA as shown in D.

Acquisitions are preceded by an overview image acquisition (Figure S1). Low magnification images are acquired in single-beam mode (a single beam is selected through the variable aperture (Figure 1)), mapping the locations of the sections (Figure 3A). The overview images also help define the location for FAST-EM calibrations, which are run before every acquisition. The sample must first be brought into both optical and e-beam focus. An optical autofocus routine is performed (Figure 3B), which moves the sample stage in *z* to position the sample in the focal plane of the optical objective lens, while recording the spot profile on the diagnostic camera. The optical focus is subsequently monitored during image acquisition. After the optical focus is determined, the system is again switched to single-beam mode and the electron beam lens and stigmator alignment, focusing and astigmatism correction are performed by the user. Because the common crossovers of all beams are positioned in the objective lens and stigmator, the alignments for the single beam directly apply to all other 63 beams.

After the correct settings are found for the electron optics, three additional optical calibration steps must be performed in multibeam-mode prior to imaging to ensure that seamless, homogeneous multibeam field-of-views (*fields*) are produced from the individual 64 beamlet images (*cells*). The calibration steps are fully automated in the microscope acquisition software, but the location on the sample where these are performed must be defined by the user (Figure S2). The first calibration step aligns the multiprobe to the MPPC detector array and determines the scan orientation (not shown). The second calibration determines a digital dark offset and gain value for each MPPC to homogenize the intensities between individual beamlets (Figure 3C). This is necessary because individual MPPCs have slightly different gain factors. The last calibrations step then determines the translation between individual cells in order to produce a seamless image from 64 beams. The microscope scans a 900 × 900 pixel area per beamlet (100 pixel overlap) on the biological sample. The stitching is then determined by finding point matches in the overlap area and minimizing the distance between them in adjacent beam images.

Finally, regions of acquisition (*ROAs*) are defined by the user on the overview images with the ROA tool (Figure 3D, Figure S1). When the acquisition is initiated by the user, the microscope software determines the amount of fields required (with some overlap between fields) to fully image an ROA, and all defined ROAs are then automatically acquired by *mosaicking* with stage increments of 24 µm (Figure 3D and E). This produces a set of 2D images for all ROAs in the specimen (a single acquired ROA is referred to as a *megafield*). The raw images (900 × 900 pixel per beamlet) are real-time processed into seamless images of 6400 × 6400 pixels and transferred to a local storage server. On user request, the unprocessed raw images (7200 × 7200 pixels) can be saved instead.

### 2.3 Image processing of large-scale FAST-EM datasets

We implemented an image processing workflow for FAST-EM datasets based on published software libraries for large volume reconstructions. The workflow is designed to be flexible, since acquisitions on large areas may lead to inconsistencies in data quality due to local variation of the sample preparation. Acquisition and image processing can be performed on individual sections in case reacquisition is needed because of errors. Additionally, visualization of intermediate image processing steps is incorporated to identify problems and perform qualitative assessment of the results. This also allows for reprocessing with optimized parameters.

**Figure 4:**
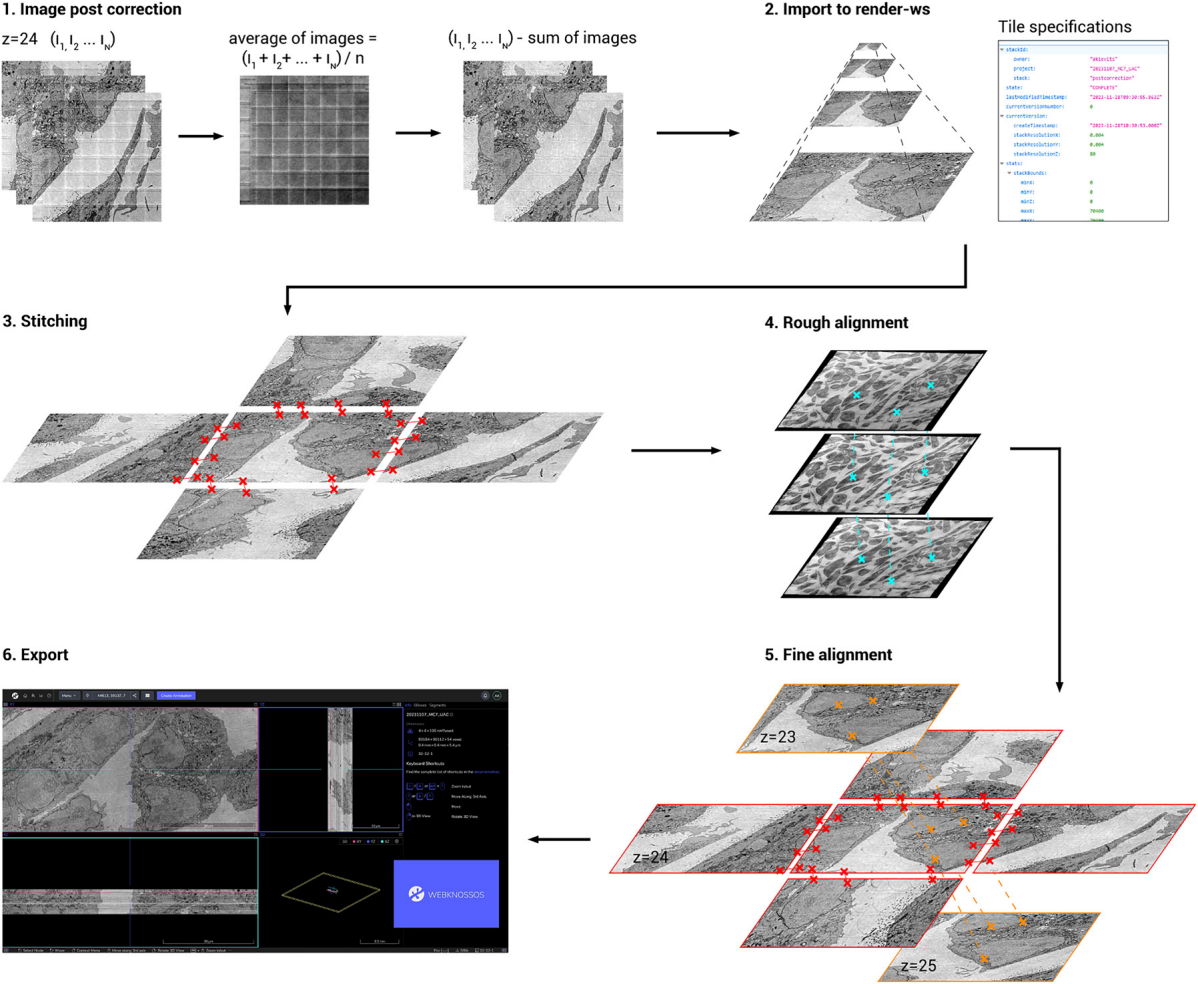
Image processing workflow. Images are first post-corrected for beam artifacts using an average correction image. Images are then imported into render-ws, which generates an image pyramid (Mipmap) and sets the tile specifications from the metadata. Tile pairs (neighbors in *xy*) are determined in the same ROA and stitched. The stitched megafields are downscaled and point-correspondences are computed to roughly align the stack in 3D. Tile pairs determined from the roughly aligned stack (neighbors in *z*) are fine aligned in 3D. Finally, the aligned stack is exported to WebKnossos for viewing in 3D.

Images are first post-corrected to remove intensity differences remaining after calibration and produced from the overscan (Figure 4). Per ROA, the average of all images is calculated and then subtracted from all images in the specific ROA. Fields that contain artifacts are detected by an outlier detection algorithm and are excluded from the average image (see methods for implementation details). This procedure is designed to fail when a ROA contains many artifacts such as caused by dirt particles on the section, as this would produce a correction image that is biased by high-contrast features. In this case, the correction is performed using the correction image from the nearest section in *z* where post-correction succeeded.

The resulting post-corrected images and their metadata are imported to a local instance of render-ws,1
https://github.com/saalfeldlab/render. which assigns a unique identifier to every image and keeps track of its individual transformations during downstream post-processing [20]. Render-ws also saves the point-match correspondences found for each image during stitching and alignment. Tile pairs in the same ROA are then defined based on the metadata, and stitched into a montage based on point-correspondences sought in the overlap region. The images are then aligned in 3D using a two-step approach, where first an approximate rough alignment is determined from downsampled montages to find neighboring images in *z*, followed by a tile-to-tile fine alignment. The final result is then exported to disk and uploaded to WebKnossos [21] to be processed or analysed further.

### 2.4 Large-scale and volume acquisitions with FAST-EM lead to consistent high-resolution images

**Figure 5:**
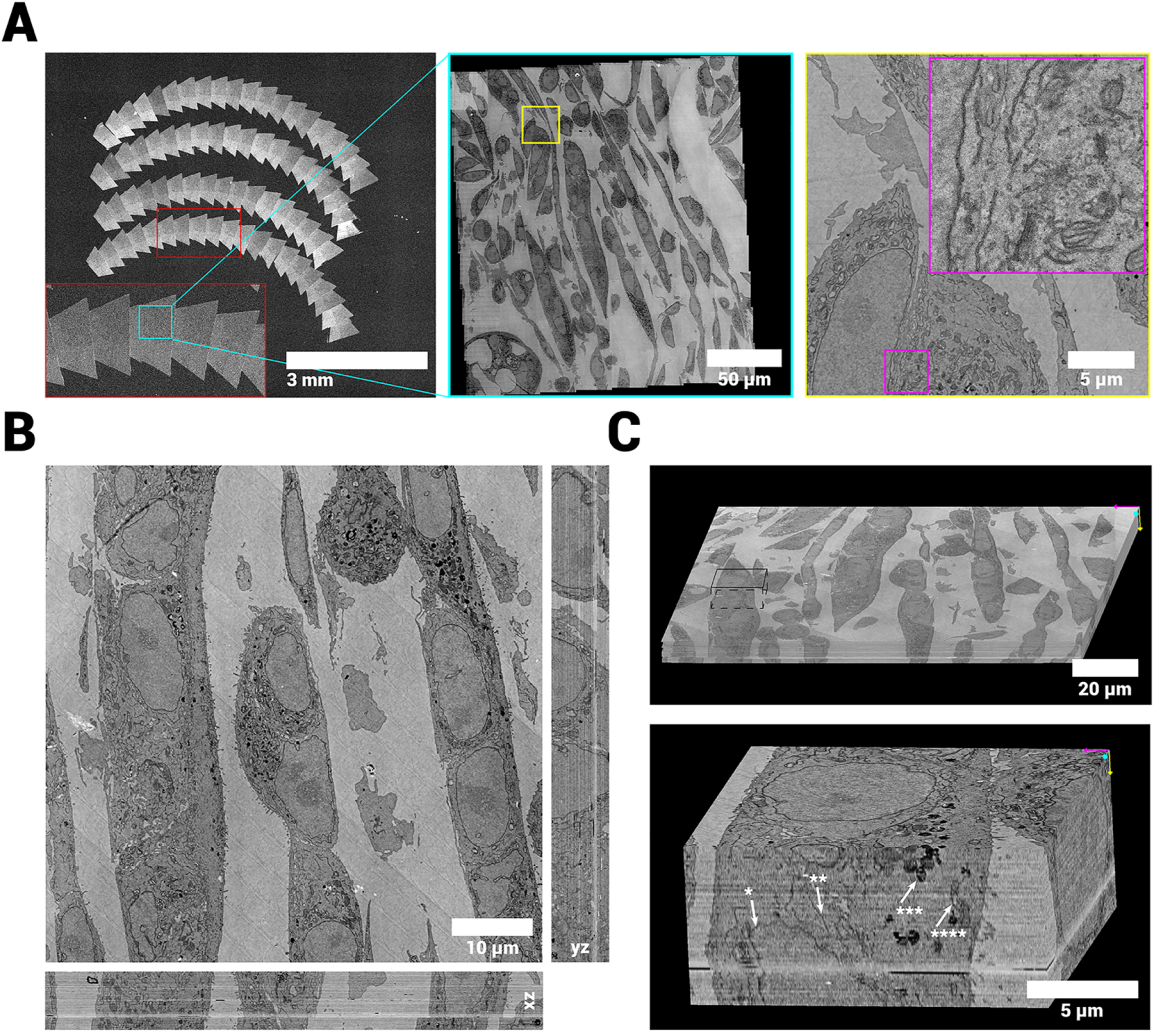
vEM reconstruction of cultured MCF-7 cells. A Overview images of sections, showing a zoom in on a single ROA, a single field and a single cell respectively. B Aligned volume reconstruction from 72 100 nm serial sections showing the orthogonal reslices through the center of the stack (*xz* and *yz*). C Volume rendering of the full (continuous) stack. Inset shows smaller subvolume at 8 nm/pixel resolution with arrows pointing at structures of interest (star indicators: *=nuclear membrane, **=endoplasmatic reticulum, ***=lysosome, ****=Mitochondrium). The data quality and alignment is consistent throughout the stack. The complete 3D dataset at full resolution is available via Nanotomy.2
http://www.nanotomy.org/OA/Kievits2024MIM/index.html.

We prepared several samples for array tomography with 100 nm section thickness, including tissues and cell cultures, imaged them with FAST-EM, and reconstructed the volumes using the implemented image processing workflow (Figure 5A, Figure S3A and Table 1). Samples prepared with the ferrocyanide-reduced osmium-thiocarbohydrazide-osmium (rOTO) protocol [22] resulted in images with decent contrast. Cells stained with neodymium acetate [23] as opposed to uranyl acetate demonstrated remarkably similar contrast, indicating that the rOTO protocol is a suitable basis for preparing samples for FAST-EM.

**Table 1:** Datasets presented in this publication. The voxel size and field size are 4 × 4 × 100 nm and 6,400 × 6,400 pixels respectively for all datasets. The tile overlap was increased for several ROAs in the MCF-7 NdAc dataset to ensure sufficient overlap.

Dataset	Figure	Dwell time (µs)	No. of sections	Acquisition time (hours)	Effective through-put (MPx/s)	Tile overlap (pixels)	Raw data size (GB)	ROA size (µm)
MCF-7 NdAc	5	10	72	16.7	2.75	400, 640	489.6	192 × 192
MCF-7 UAc	6, S4	20	54	29.7	1.82	400	573.8	240 × 240
Rat pancreas	S3, S5	10	44	–	–	400	74.8	96 × 96

Little residual intensity variations can be seen in the *xy* plane of the data, indicating that the image post-correction procedure is consistent. The effect of residual intensity differences after calibration and beam overscan is seen mainly in empty resin, where no biological features are found. The intensities and resolution are also consistent throughout the image stack.

The proportion and resolution of the data sets make it possible to trace a large number of subcellular structures and cell organelles throughout the volume (Figure 5B and C, Figure S3B and C). The axial resolution allows identification of some organelles in the *xz* and *yz* planes (Figure S4A). Nuclear membranes, mitochondrial membranes and cristae, ER, Golgi stacks and lysosomes can be reliably identified at full data resolution (Figure S4B).

By default, the alignment is solved for a set of similarity transformations (rotation, translation and scaling) on the joint set of point-correspondences between images in the same *z*-layer and between *z*-layers. This produced consistent global results, but would not always produce accurate local alignment. More elaborate transformations (i.e. full affine, polynomial transforms) lead to a higher local alignment precision, but would not always yield a globally consistent result. The initial fine alignment was therefore refined with optical flow [24], which is able to determine the fine alignment using elastic deformations while maintaining the original geometry of the biological sample [25]. This improved the local alignment, supposedly due to the algorithm being able to compensate non-linear deformations introduced during sectioning which cannot be accounted for by rigid and scaling transformations alone. A single misalignment can be seen (Figure 5B and C); on closer inspection of the data, however, this misalignment appears to originate from a discontinuity in the dataset which coincides with a transition between ribbons. This type of misalignment was not observed in other datasets (Figure S3, Figure S4). Therefore, this result is attributed to section loss during the preparation of the ribbons.

### 2.5 Leverage of automated segmentation demonstrates applicability of FAST-EM

FAST-EM data can be streamed efficiently in 3D using the WebKnossos viewer. Using WebKnossos’ Python API, it is possible to access and load arbitrary views of the data at different zoom levels, which can be directly visualized and annotated in tools like FIJI or Napari [26] or further processed using popular tools for image analysis [27], [28].

**Figure 6:**
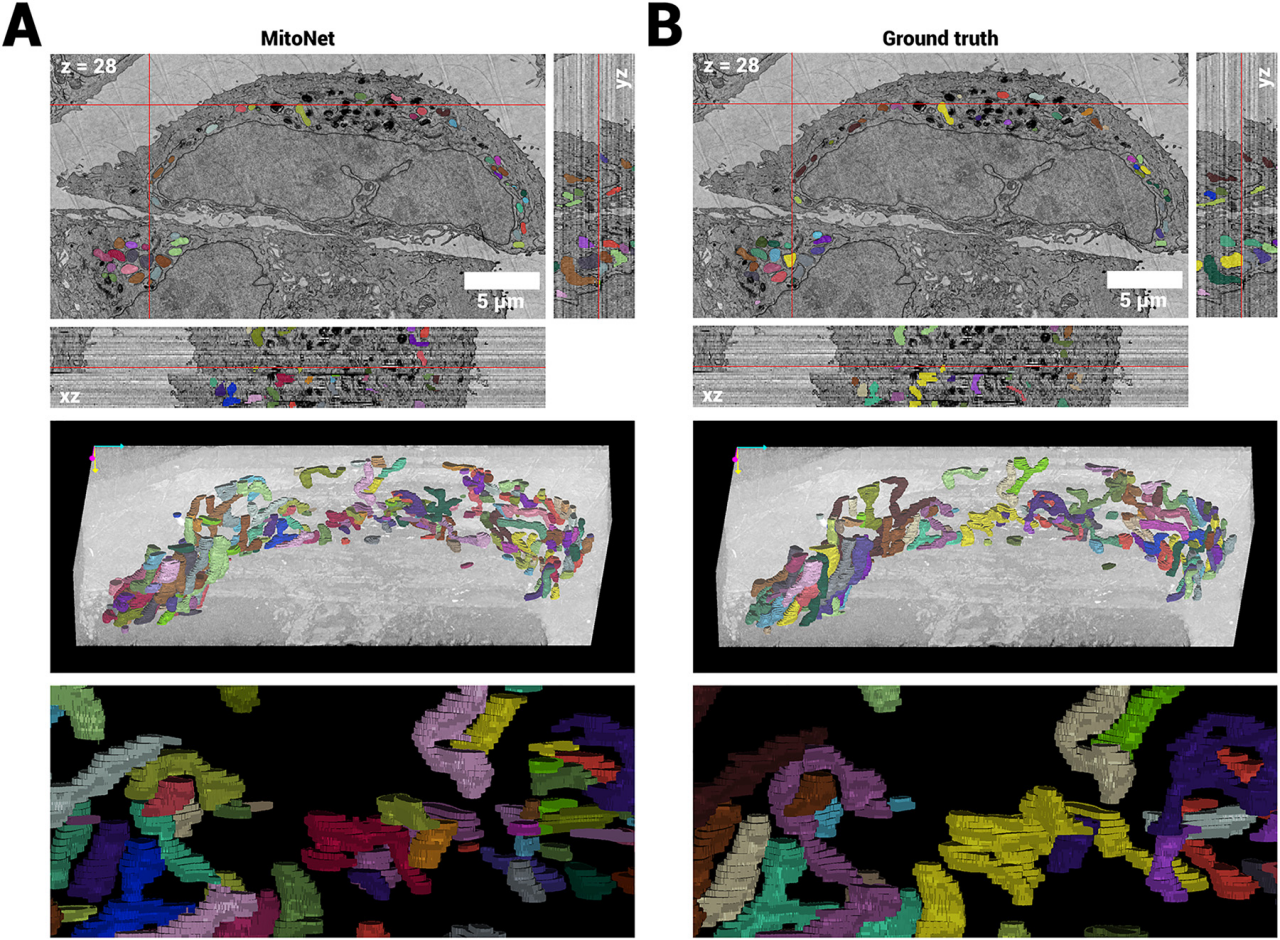
Automatic instance segmentation of mitochondria in FAST-EM data using MitoNet [29]. A MitoNet predictions on subset of data, showing the orthogonal slices at the locations indicated by the red cross, and 3D renderings in Napari. B Ground truth annotations of mitochondria from the same volume. The predictions show qualitative agreement with the ground truth, although some split errors can be observed.^2^

All mitochondria were automatically segmented with MitoNet [29] (available as the Empanada plugin in Napari) to demonstrate the usability and applicability of analysis tools developed for other vEM modalities and datasets to OSTEM-detection based FAST-EM data. MitoNet is a generalist convolutional neural network architecture for segmenting mitochondria trained on a diverse training dataset. Notably without retraining nor finetuning the network architecture on FAST-EM data, 3D inference with MitoNet produced qualitative good results, where it would recognize a large portion of the ground truth annotated mitochondria in MCF-7 cells prepared with a modified FIB-SEM staining protocol (Figure 6A). MitoNet was also applied to rat pancreas tissue, which yielded similar agreeable results (Figure S5A). Mitochondria in the MCF-7 cells appeared to have complex, elongated ultrastructure, whereas the rat pancreas datasets presented mitochondria with a more diverse collection of elongated as well as spherical mitochondria.

Several hundreds of mitochondria were manually annotated in a subset of the MCF-7 cell and rat pancreas datasets to assess the quantitative performance of MitoNet on FAST-EM data (Figure 6B and Figure S5B). The semantic IoU (intersection over union), F1 and AP (average precision) scores were then determined on both the originally aligned data and the realigned data with optical flow, to investigate the effect of alignment precision on the segmentation quality (Table 2). MitoNet demonstrated IoU scores comparable to benchmark datasets obtained using vEM modalities based on other electron detection techniques (e.g., HeLa with IoU: 0.791, F1@50: 0.728 and AP@50: 0.573 and *C. elegans* with IoU: 0.60, F1@50: 0.483 and AP@50: 0.318, both FIB-SEM datasets), but overal lower F1 and AP scores. Notably, the IoU scores on the rat pancreas dataset were lower (0.136 point) than for the MCF-7 cell dataset, but the F1@50 and AP@50 scores were higher (0.129 and 0.11 point respectively). The realignment of the data with optical flow did not overall influence the IoU scores, indicating no effect on semantic segmentation performance of the model. However, it did have a noticeable positive effect on F1 and AP scores (F1@50 0.266 and 0.159 point increase, AP@50 0.178 and 0.124 point increase for MCF-7 and rat pancreas, respectively). This indicates that the network is able to predict complete mitochondria more effectively on the data realigned with optical flow than on the original fine aligned data. This suggestion was substantiated by a reduced amount of false positives for both realigned datasets. A slightly larger improvement in F1 and AP scores was noted for the cell dataset than for the rat pancreas tissue.

**Table 2:** Performance metrics for MitoNet 3D instance segmentation on FAST-EM data. **IoU**: Intersection over union (Jaccard index). **F1@50/75**: F1 score at 0.5/0.75 IoU threshold. **AP@50/75**: Average Precision at 0.5/0.75 IoU threshold.

Dataset	# GT mitos	IoU	F1@50	F1@75	AP@50	AP@75
MCF-7 UAC fine aligned	97	0.778	0.133	0.078	0.071	0.041
MCF-7 UAC realigned	97	0.770	0.399	0.196	0.249	0.109
Rat pancreas fine aligned	217	0.615	0.379	0.080	0.235	0.042
Rat pancreas realigned	217	0.644	0.528	0.142	0.359	0.076

### 2.6 Scaling up acquisitions

FAST-EM has been designed for large volume acquisitions of tissues and cells. Of interest therefore are the scalability of the acquisition and image processing to a large volume and the expected acquisition and reconstruction times. The recent introduction of high-throughput vEM modalities has in turn required the development of image processing workflows capable of handling petabyte scale datasets [20], [30], [31]. Such data sets are not yet available from FAST-EM, but the tools that are implemented in the image processing workflow have been demonstrated on millimeter-sized volume datasets. Therefore, the workflow should be scalable to larger volumes, provided that the necessary computational infrastructure is available.

**Table 3:** Estimated acquisition times for FAST-EM of a 500 × 500 × 50 μm^3^ volume from 500 serial sections, compared to a single-beam SEM [32], beam-deflection GridTape TEM (bd-TEM [6]), and automated tape-collecting ultramicrotomy combined with MuliSEM imaging (ATUM-MultiSEM, [33]). For FAST-EM, with the ROA placement precision of approximately one field, a padding of one row or column of fields on each ROA edge is assumed. Numbers indicated for FAST-EM are based on 10 µs dwell time as used in this study, and in brackets for 2 µs, which is feasible for brain tissues (data not shown) or when the beam current limitation in the current early adopter system is lifted.

	SEM	FAST-EM	bd-TEM	ATUM-MultiSEM	Unit
Dwell time	1	10 (2)	0.05		µs
Pixel size	4	4	3.6	4	nm
FoV acquisition time	16.78	8.10 (1.62)	0.040	0.6	s
Stage overlap	10	6.25	10	6	%
Stage time per FoV	2	0.52	0.055	1	s
Per section overhead	–	52	132	36	s
FoVs per section	1,156	529	81	50	
Time per section	21,706	4,842 (1,414)	171	116	s
Total time	3,015	672.5 (196.4)	23.7	16.3	h
Sustained throughput	0.72	3.23 (11.05)	91.48	133.55	MPx/s

FoV, field-of-view.

The maximum volume for FAST-EM array tomography is restricted to the number of serial sections that fits on a single 14 × 14 mm scintillator. The sample can be divided over multiple scintillators, but this requires interruption of the sectioning process and therefore involves a significant risk of section loss. A sample area of 1 mm^2^ (typical in bd-TEM and MultiSEM combined with ATUM) would lead to a very limited number of sections on a single scintillator; a section size of 500 × 500 μm^2^ allows for a larger *z* dimension. An estimated 500 sections of this size can fit on a single scintillator with a high packing density, which would also be close to the practical number of sections possible with array tomography. Assuming a section thickness of 100 nm, this yields a volume of 500 × 500 × 50 μm^3^. The total estimated FAST-EM acquisition time is then computed for this sample volume and for other vEM modalities (single-beam SEM, bd-TEM and ATUM-MultiSEM, Table 3) using the reported acquisition and overhead times for a single FoV and section (if needed, corrected for the section size). The sustained throughput is then defined as the number of pixels in the volume divided by the expected acquisition time. Overhead for sample exchange, setting up the acquisition (Figure S6A) and reacquisitions are not included in this calculation. Additionally, the reconstruction time was calculated assuming the resources available on the dedicated storage server of FAST-EM (Table S1).

The calculation yields a sustained throughput for FAST-EM of 3.23 MPx/s at a 10 µs dwell time, and 11.05 MPx/s at a 2 µs dwell time. This shows that the early-adopter FAST-EM is already significantly faster than a single-beam setup (0.72 MPx/s), but the throughput is still an order of magnitude lower than bd-TEM and ATUM-MultiSEM (91.48, 133.55 MPx/s respectively). Notably, for a dwell time of 2 µs and 10 µs, the majority of the acquisition time is spent on scanning (Figure S6B). The estimated reconstruction time (54.8 days) is longer than the acquisition time (28 days at 10 µs dwell, 8.2 days at 2 µs dwell). This is because the compute resources of the dedicated storage server of FAST-EM are limited (40 CPU cores).

## 3 Discussion

FAST-EM is compatible with the existing rOTO protocol, as exemplified by both the cellular and tissue samples that were imaged and reconstructed here. Our results also show that substitution of uranyl acetate by neodymium acetate [23] yields images with similar contrast for cells. A thorough investigation and comparison of different sample preparation protocols and their effects on image contrast obtained with the OSTEM detector in FAST-EM is a subject of ongoing research.

Scintillator substrates designed for FAST-EM are demonstrated to support conventional serial-section array tomography approaches [18], [19]. The substrates provide a large unobstructed area for imaging similar to silicon wafers or ITO-coated coverslips. If a single substrate is not sufficient, multiple substrates can be used for a single sample. This, however, requires interruption in sectioning and thus may not be feasible in practice. The substrates are in principle compatible with alternative section collection techniques for volume EM, such as tape-based collection (ATUM) and magnet-based collection (MagC [34] and GAUSS-EM [35]). In practice, however, the compatibility with ATUM seems limited by the low fill factor and the transparency of the tape (we note that electron-transparent tape is available [8] but would need to be tested for compatibility with OSTEM detection in FAST-EM). The production and future use of larger scintillator wafers to accommodate more sections is likely possible, which would favour the combination with magnet-based collection, in which the sections are deposited directly on the substrate in random order and orientation as opposed to ordered ribbons.

Recently, nanoscale light microscopy-based imaging has been achieved with effective throughput rates comparable to vEM, in combination with molecular labeling [36]. Indeed, the use of correlative (light) microscopy in combination with volume electron microscopy (vCLEM) can yield biological specificity or facilitate region-of-interest selection for FAST-EM. The substrates used currently in FAST-EM, ce:YAG, are incompatible with integrated CLEM [37] or in-resin CLEM [38] as they are luminescent at commonly used excitation wavelengths for fluorescence microscopy, thus generating significant background noise. However, transparent scintillator materials can be used instead, provided that they yield sufficient light output for EM.

Overview images produced in single-beam mode provide sufficient guidance for defining ROAs and pave the way for future automatic identification of sections. While the definition of ROAs is currently still manual and limited to rectangles, we expect future software updates to be compliant with arbitrary ROA shapes or even automatic mapping. With incorporation of focus and astigmatism routines, the image acquisition procedure could be fully automated.

The post-correction of the images is a necessary but effective method for removing intensity differences caused by overscan or parking of the beams, and residual intensity differences remaining after digital offset and gain calibration and imperfect alignment of the multiprobe. The post-correction reduces or completely removes intensity differences that appear for each cell position in every field. However, there are some inconsistencies in the data that cannot be corrected for with this procedure: beam exposure artifacts in re-acquisitions of ROAs; differences in intensity distributions between sections (since the correction is performed in-plane); errors in the stitching of adjacent beamlets and sample tilts which cause large deviations from the calibration settings. Note that the latter two can be avoided by bypassing calibration step 3 through saving the raw images (at the cost of extra post-processing), and careful placement of the sample on the holder to prevent tilts. Notably, the post-correction failed to remove certain diagonal stripe artifacts appearing for each cell image in the dataset presented in Figure 6. The artifacts are proportionally more expressed in empty resin than in tissue or cells, and thus do not appear homogeneously through the dataset, which explains why they are not fully removed. The exact cause of the artifacts is a topic of investigation, but is currently attributed to sample damage from e-beam exposure.

Segmentation of mitochondria with MitoNet demonstrated similar IoU scores to benchmark datasets from other vEM modalities. This shows that MitoNet is capable of generalizing to FAST-EM datasets, and further establishes that FAST-EM data resembles data from other vEM modalities, both to a microscopist’s eye and a neural network. Instance segmentation scores were overall lower than for the MitoNet benchmark datasets. This can be explained by the anisotropic voxel size; whereas the data reported here has a *z* resolution of 100 nm, most MitoNet benchmark datasets have higher *z* resolution, with several having isotropic voxels. Therefore, decreasing the section thickness is expected to lead to higher instance segmentation performance. Furthermore, Empanada offers tools to finetune MitoNet on images of specific datasets, which may improve semantic and instance segmentation scores on FAST-EM data.

The effective throughput for the datasets reported in this publication includes the total time spent on acquisition set-up, reaquisitions and monitoring image quality. It is difficult to calculate these numbers for other vEM modalities, especially since these would depend on the specific sample that is imaged. Therefore, we used the sustained per-section throughput of FAST-EM calculated at both 2 µs and 10 µs dwell time (11.05 and 3.23 MPx/s respectively, including overhead from stage translations and calibrations) in the comparison with other vEM modalities. The per-section throughput of FAST-EM array tomography and *z* resolution are lower than for bd-TEM and ATUM-MultiSEM. However, the early adopter FAST-EM system still has several restrictions. The beam current is fixed at 0.4 nA. Future updates will allow a larger beamlet current without significant compromise on resolution (up to 1 nA per beamlet is possible), allowing for similar contrast and SNR at shorter dwell times. Likewise, the landing energy is fixed at 5 keV, which is a suboptimal energy for sections thinner than 100 nm. For a specific sample composition and section thickness, there exists an optimal landing energy [15]. Furthermore, the landing energy affects the crosstalk between optical signals and the image resolution; at higher keVs, the crosstalk is bigger due to the larger interaction volume of the e-beam and hence more intensity in the long-range tails of the optical spot profile of each beamlet. At lower keVs, the image resolution may be compromised due to increased chromatic aberrations. Future updates will allow tuning of the landing energy with respect to the sample composition and preparation, leading to the best possible contrast and SNR.

Pixel dwell times will be further reduced through optimization of the optical system and scintillator supply. Optimization of calibration procedure times and stage settling times has not been performed and can lead to signification reduction of overhead times. Future instrumentation development will focus on modeling and subsequent optimization of the OSTEM detector, leading to shorter possible dwell times. Another point of improvement is the beam pitch. To increase the pitch, a redesign of the electron-optical column is required. The pixel size is set by the magnification of the optical system; larger pixel sizes subsequently change the pitch and therefore the distance between spots on the detector. Currently, the magnification of the optical system is fixed. To reach an optimal dwell time for an aimed-for resolution in FAST-EM, all aforementioned factors should be considered in subsequent design improvements.

We have demonstrated a workflow implementation for volume electron microscopy using a commercially available multibeam scanning transmission electron microscope, FAST-EM. The applicability of FAST-EM to several diverse biological samples is shown. Multibeam OSTEM detection is shown to be compatible with community tools for volume alignment, reconstruction and segmentation, even when these algorithms have been developed using data obtained with other EM modalities. The data is released to the community as benchmark for future projects or for further analysis. Cellular organelles have major roles in regulating cellular metabolism and homeostasis, and it is crucial to understand their structure and function relationships. Overall, FAST-EM proves itself as a promising tool for analysis of cellular as well as subcellular organelle ultrastructure in 3D by providing high-throughput quantitative measurements. We envision FAST-EM will be further utilized in the future to systematically address how organelle ultrastructure is altered in relation to certain mutations, oncogenes, drugs and other environmental factors.

## 4 Materials and methods

### 4.1 Sample preparation

Rat pancreas samples were prepared as previously described [15], where uranyl acetate was replaced with spun-down 4 % neodymium acetate [23]. Briefly, tissue was aldehyde fixed, vibratome sectioned, subjected to reduced osmium-thiocarbohydrazide-osmium (rOTO) post-fixation (1 % osmium tetroxide, 1.5 % potassium ferrocyanide and 4 mM calcium chloride in 0.1 M sodium cacodylate buffer [39]), *en bloc* stained with neodymium acetate followed by lead aspartate, dehydrated and flat embedded in EPON between ACLAR sheets.

Sample fixation and staining and embedding of MCF7 cells was achieved similar to as reported before [40], [41]. In short, samples were fixed with 2.5 % glutaraldehyde and 2 % paraformaldehyde in 1× PHEM buffer, and poststained with 1 % osmium tetroxide, 1.5 % potassium ferrocyanide in 0.065M PHEM for 2 h at 4 °C, followed by 1 % thiocarbohydrazide (Sigma) for 20 min at RT, 1 % OsO4 in ddH2O 30 min at 4 °C, 1 % uranyl acetate (or 4 % neodymium acetate) at 4 °C overnight, and Walton’s lead aspartate (pH 5.6) for 30 min min at 58 °C. Samples were then dehydrated and infiltrated with EPON resin.

### 4.2 Specimen preparation

For the rat pancreas sample, molybdenum thin-film coated yttrium aluminum garnet scintillator (ce:YAG) plates were received from Delmic B.V. For the MCF-7 cells, ce:YAG was ordered from Surface Preparation Laboratory (SPL). RF magnetron sputter coating was performed on the SPL scintillators in-house with an AC450 (Alliance Concept) with 150 W RF at 3 µbar for 32 s to achieve a layer of 30 nm molybdenum.

The scintillator substrates were submersed in the water bath before sectioning. The tissue block was first trimmed to a trapezoidal block face. The presence of tissue or cells in the surface of the block face was verified by cutting a semithick section and staining this with toluene blue. Glue was then applied at the top and bottom of the block face to ensure the serial sections would stick, facilitating the formation of long ribbons. A single long ribbon of ultrathin sections (100 nm) was then cut using a Leica UC7 (MCF-7 cells) or Leica ARTOS 3D (rat pancreas). The ribbons were split into 3 or 4 smaller ribbons. The water level was then gently lowered to deposit the ribbons on the substrate. No additional coating was performed before imaging.

### 4.3 Electron microscopy

The sample was mounted on the FAST-EM sample holder using 60 µm-thick Kapton tape on two sides opposite of the sample. The sample was then pumped to high vacuum and acclimatized for at least 12 h. An optical focus calibration was then performed near the middle of the scintillator. Overview images were made of the sample in single-beam mode using the T1 detector (backscattered electrons) at 1.5 mm horizontal field width to facilitate the selection of ROAs. Electron beam alignment was performed in single-beam mode at 60,000× magnification, and the beam was focused and corrected for astigmatism. This was followed by the FAST-EM specific calibrations, which were performed once per volume acquisition, as close to the middle of the scintillator as possible. Calibrations 1 (multiprobe alignment) and 2 (digital gain and offset) were run on a part of the scintillator where no sample was present. Calibration 3 (cell translation) was performed on a region of the sample not part of the final ROA, with continuous features (i.e. biological structures) throughout a region approximately the size of a single field. All acquisitions were performed with a 5 keV beam energy, 0.4 nA beam current and 4 nm pixel resolution. A dwell time of 10 µs was used for both the rat pancreas and MCF-7 cell specimens stained with neodymium acetate, and 20 µs for the MCF-7 cell specimen stained with uranyl acetate. All procedures except for the electron beam focusing are implemented in ODEMIS,3
https://github.com/delmic/odemis. which is open source software. The source code for the calibrations is closed source.

### 4.4 Serial data acquisition

ROAs were defined on adjacent sections. Each ROA position was manually verified and corrected if necessary using the single beam mode, centering the ROA position on features continuous in serial sections such as outlines of cells or contours of tissue. This ensured that the ROAs would be aligned with an accuracy of roughly a single field (24 µm). No scan rotation was applied to correct for the ribbon rotation, as this is not available in the early adopter model. Focus and astigmatism were manually corrected every 5 or 10 sections, or at the start of a new ribbon, which was performed in the middle section.

### 4.5 Image post-correction

Image post-correction was performed by averaging all images in a single ROA and then subtracting the average image from every other image. This effectively removes residual intensity differences that are a result of scan overlap, beam flybacks and calibration errors. Outlier fields (i.e. with a deviating histogram) were excluded from the averaging. Outliers are detected using the Median Absolute Deviation, i.e.:

(1) $$MAD=median\left(\left|X_{i}-\tilde{X}\right|\right)$$

where $\tilde{X}$ is the median of the 1st percentile of selected images. Images are flagged as containing artifacts if their histogram 1st percentile deviates from the median percentile:

(2) $$corrupted=p1<\tilde{X}-a*MAD|p1>\tilde{X}+a*MAD$$

where *a* is a scaling factor that can be varied to allow for larger or smaller deviations. This effectively removes fields with an abnormal histogram from the averaging, producing an artifact-free correction image. The MED and MAD values are computed from a sample of *N* images from every ROA, and a correction image is not produced when the number of artifact-free fields falls below 20. The correction image from the nearest ROA is used to correct problematic ROAs.

### 4.6 Image processing

The image processing workflow was developed based on earlier work by [37]. After post-correction, the image data and metadata are imported into render-ws. The server has 40 CPU cores for processing, but the software can take advantage of infrastructure that is available by multithreading. The images (*tiles *in render-ws) and their respective metadata (transformations) are organized into stacks, configured as entries in a MongoDB database. Copies of the raw and post-corrected data exists on disk; only the final 3D alignment is additionally rendered to disk, whereas intermediate versions in the processing workflow are defined only by their transformations. The workflow is written in Python and JuPyter Notebook, using the render-python4
https://github.com/AllenInstitute/render-python. API to interact with render-ws, which is written in Java.

Stitching and 3D alignment of the images is based on finding matching image features in the overlap region between pairs of neighboring images with the Scale-Invariant Feature Transform (SIFT) [42]. Candidate matches detected by SIFT are filtered based on a common transformation using random sampling consensus algorithm (RANSAC) [43]. This produces a set of matched point coordinates (point matches). Using the set of point matches for all tile pairs, and after deciding on a transformation model, image transformation parameters are estimated by BigFeta.5
https://github.com/AllenInstitute/BigFeta. BigFeta solves for a set of transformations (e.g. rigid, affine) that minimizes the sum of squared distances between all point matches [44].

#### 4.6.1 Stitching

Tile pairs in 2D are identified based on the corresponding row and column indices in the file name. Point matches are then sought in the overlap region between tiles. Alignment using a translation model in BigFeta then produces a *montage*, i.e. a stitched full image of a ROA.

#### 4.6.2 3D rough alignment

Montages of adjacent tiles were first roughly aligned to find tile pairs in neighboring ROAs. Point matches are found in montages that are rendered to disk at 5 % scale. A filtering step is then performed to remove false point matches that are found on the border of the ROA. The alignment between downsampled montages is then solved, which produces a roughly aligned stack in render-ws. The transformations from this stack are then applied to the full-scale data, creating a montaged, roughly-aligned stack.

#### 4.6.3 Fine alignment

Alignment proceeds by iterating through the *z*-levels, and looking at the neighboring ROAs, sampling a cone with a radius of 0.1 times the image size to find overlapping tiles in *z*. Point matches are then sought in *z* for every tile pair. The alignment is then solved on the full set of intra-ROA and inter-ROA point matches, for a similarity transformation model, with weights given to the intra-ROA and inter-ROA matches, respectively. Regularization parameters for the transformation model were determined empirically.

### 4.7 Export

The aligned data is exported to a self-managed instance of WebKnossos [21] using the render-ws client. The data format is reduced to unsigned 8-bit and saved in .wkw format (WebKnossos data format). Segmentations are saved as 16-bit or 32-bit layers.

### 4.8 Realignment with SOFIMA

Fine aligned datasets in WebKnossos were realigned with optical flow following the approach by [24] on a single NVIDIA Tesla P100 GPU with 12 GB memory, using customized scripts. Optical flow is implemented as Scalable Optical Flow-based Image Montaging and Alignment (SOFIMA).6
https://github.com/google-research/sofima. The data sets were first cropped to a continuous volume in WebKnossos by applying a minimum projection to the full stack followed by a threshold operation. Flows were then computed from patches of 160 pixels and stride 40 on 16 nm, 32 nm and optionally 64 nm/pixel downsampled resolutions of the data. Flow fields were filtered to remove outliers. The filtered flow fields were reconciled for each position using the highest resolution flow estimate available, and the final flow was upsampled to the original resolution of the data (4 nm/pixel). A deformable mesh with Hookean springs was then fitted to the upsampled flow field. Finally, the full resolution data was warped according to the optimized mesh and exported to WebKnossos.

### 4.9 Mitochondria segmentation

Ground truth (GT) annotations of individual mitochondria were generated for the rat pancreas and MCF-7 cell dataset using the annotation tools in WebKnossos, on the originally aligned data. Mitochondria were identified based on their characteristic shape and presence of cristae, and were annotated if they were present in multiple *z* slices. Annotations were proofread by a second annotator. The GT for the SOFIMA alignment was obtained by warping the original annotations according to the deformable mesh optimized to the flow field of the data.

Mitochondria instance segmentation was performed with MitoNet [29] on data downsampled to 16 nm/pixel resolution. First, optimal MitoNet parameters for 3D instance segmentation were determined using the 2D inference tool in the empanada-napari plugin. The model was not finetuned nor retrained using ground truth annotations of FAST-EM data. For evaluation, the MitoNet predictions were first filtered to remove all mitochondria instance predictions for which no GT equivalent existed (in case of sparse annotations), while retaining all predicted pixels for the instances for which a GT equivalent existed to properly determine the IoU scores. Predicted and ground truth instances were matched using the Hungarian algorithm. IoU, F1, F1@50, F1@75, AP@50 and AP@75 scores were then calculated. For the rat pancreas, annotations consisted of two subvolumes, for which a weighted average was computed based on the number of predicted pixels in each volume.

## Supplementary Material

Supplementary Material Details

Supplementary Material Details
