# Supplementary figures and images for: FAST-EM array tomography: a workflow for multibeam volume electron microscopy
Source: Methods Microsc. 2024 Jul 11;1(1):49–64. doi: 10.1515/mim-2024-0005 (PMC11308914; doi:10.1515/mim-2024-0005)

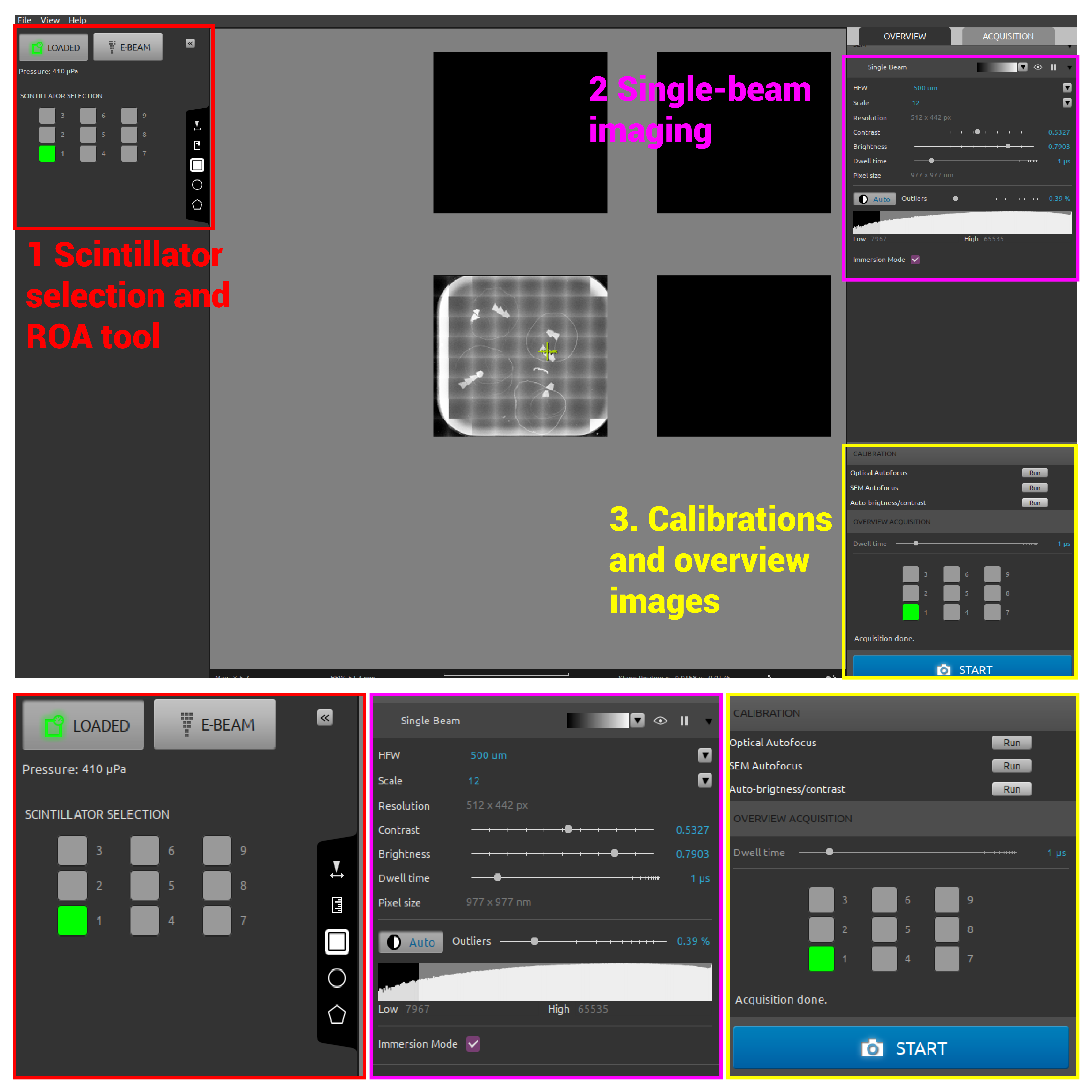

Supplement: Supplementary file 1 — Supplementary Material Details [file j_mim-2024-0005_suppl_001.zip › Supplementary_figures/S1.png]

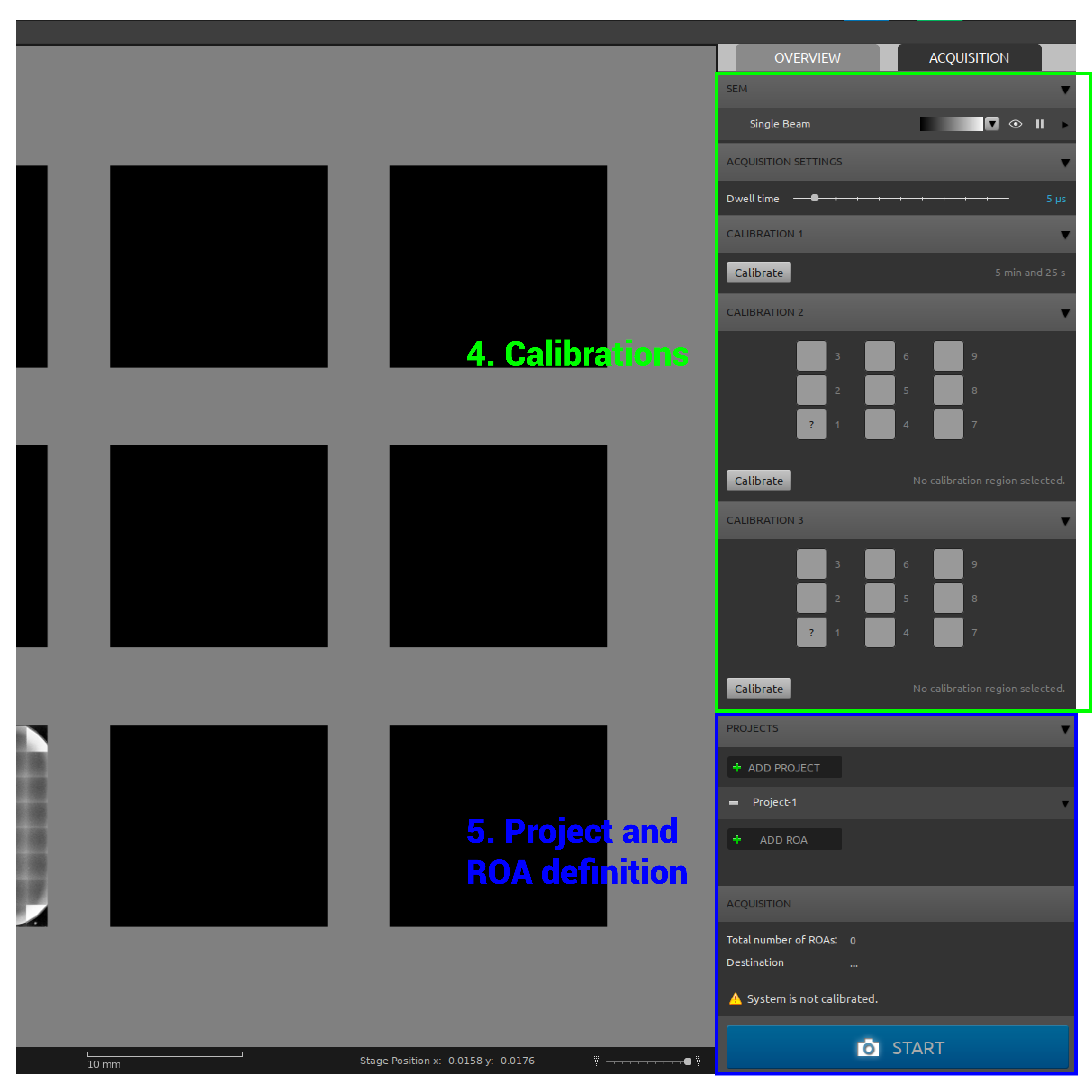

Supplement: Supplementary file 1 — Supplementary Material Details [file j_mim-2024-0005_suppl_001.zip › Supplementary_figures/S2.png]

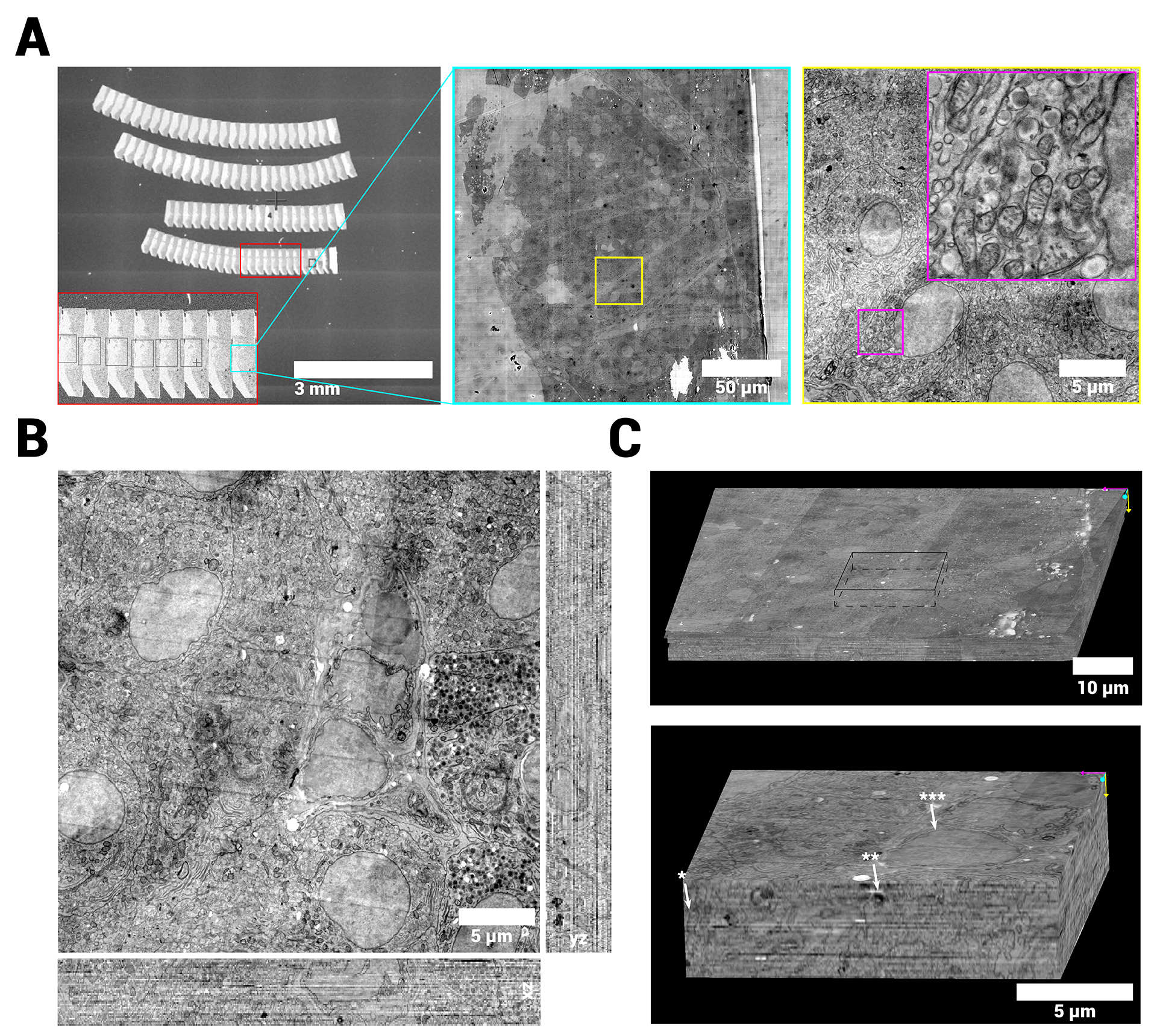

Supplement: Supplementary file 1 — Supplementary Material Details [file j_mim-2024-0005_suppl_001.zip › Supplementary_figures/S3.png]

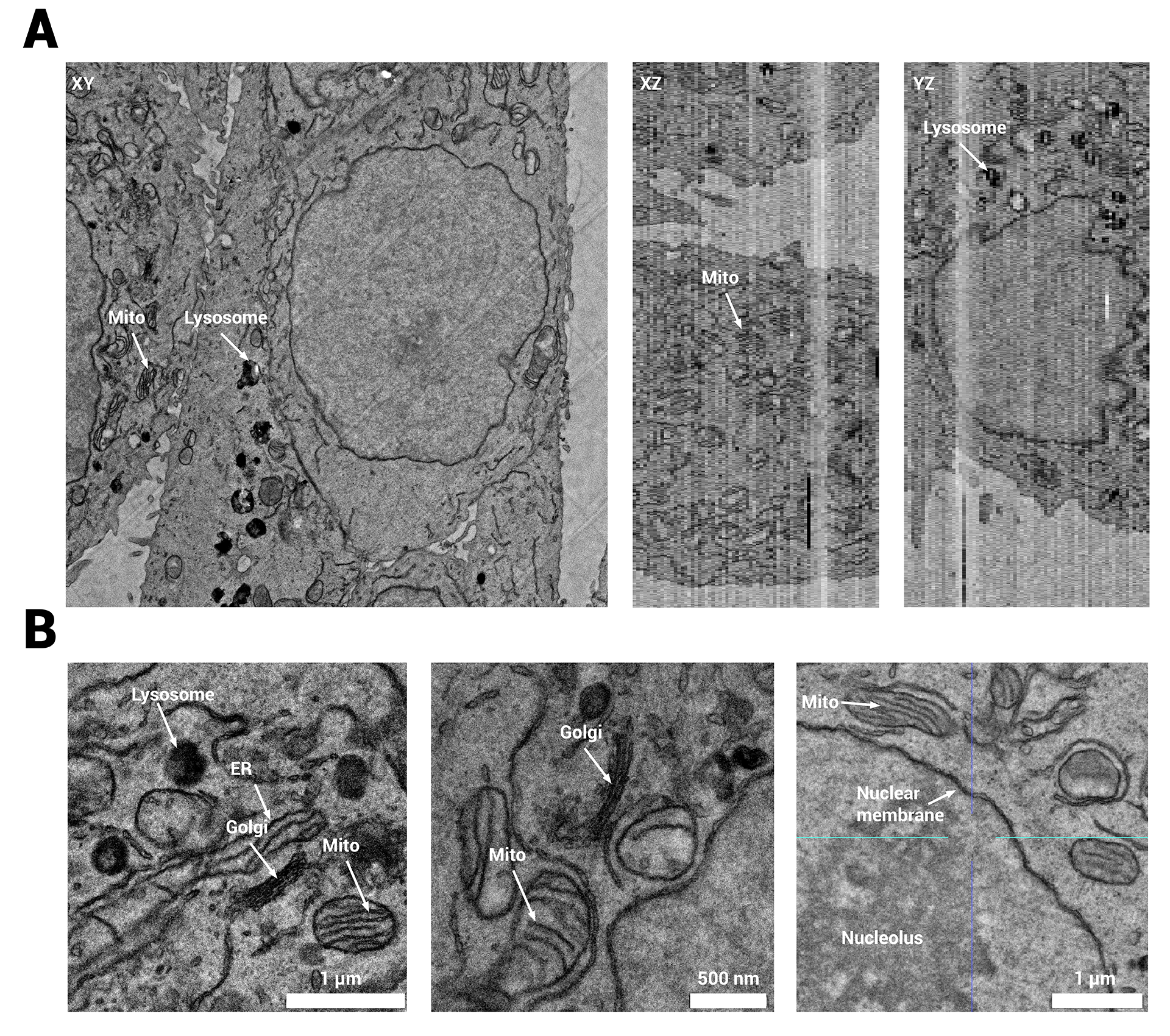

Supplement: Supplementary file 1 — Supplementary Material Details [file j_mim-2024-0005_suppl_001.zip › Supplementary_figures/S4.png]

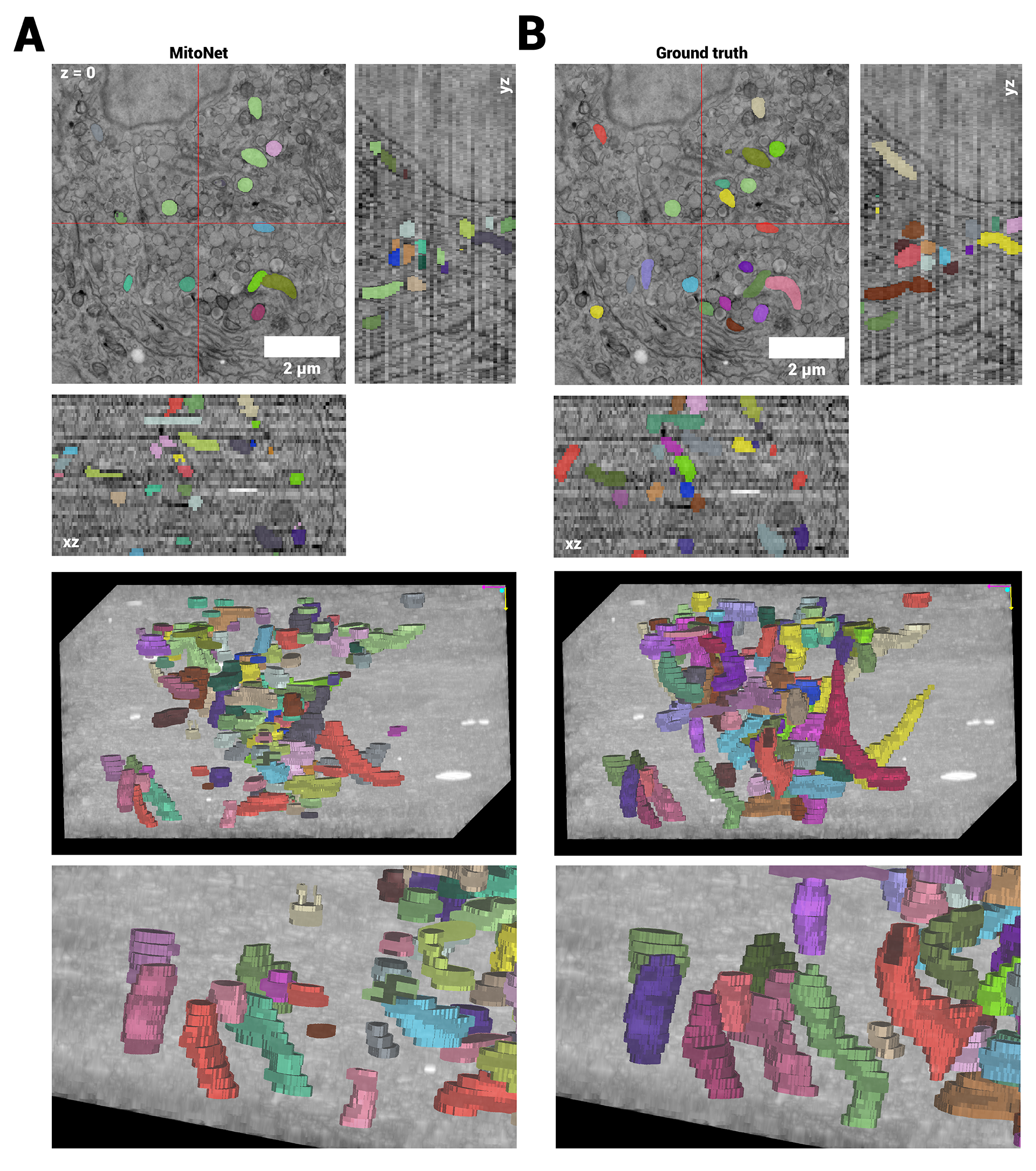

Supplement: Supplementary file 1 — Supplementary Material Details [file j_mim-2024-0005_suppl_001.zip › Supplementary_figures/S5.png]

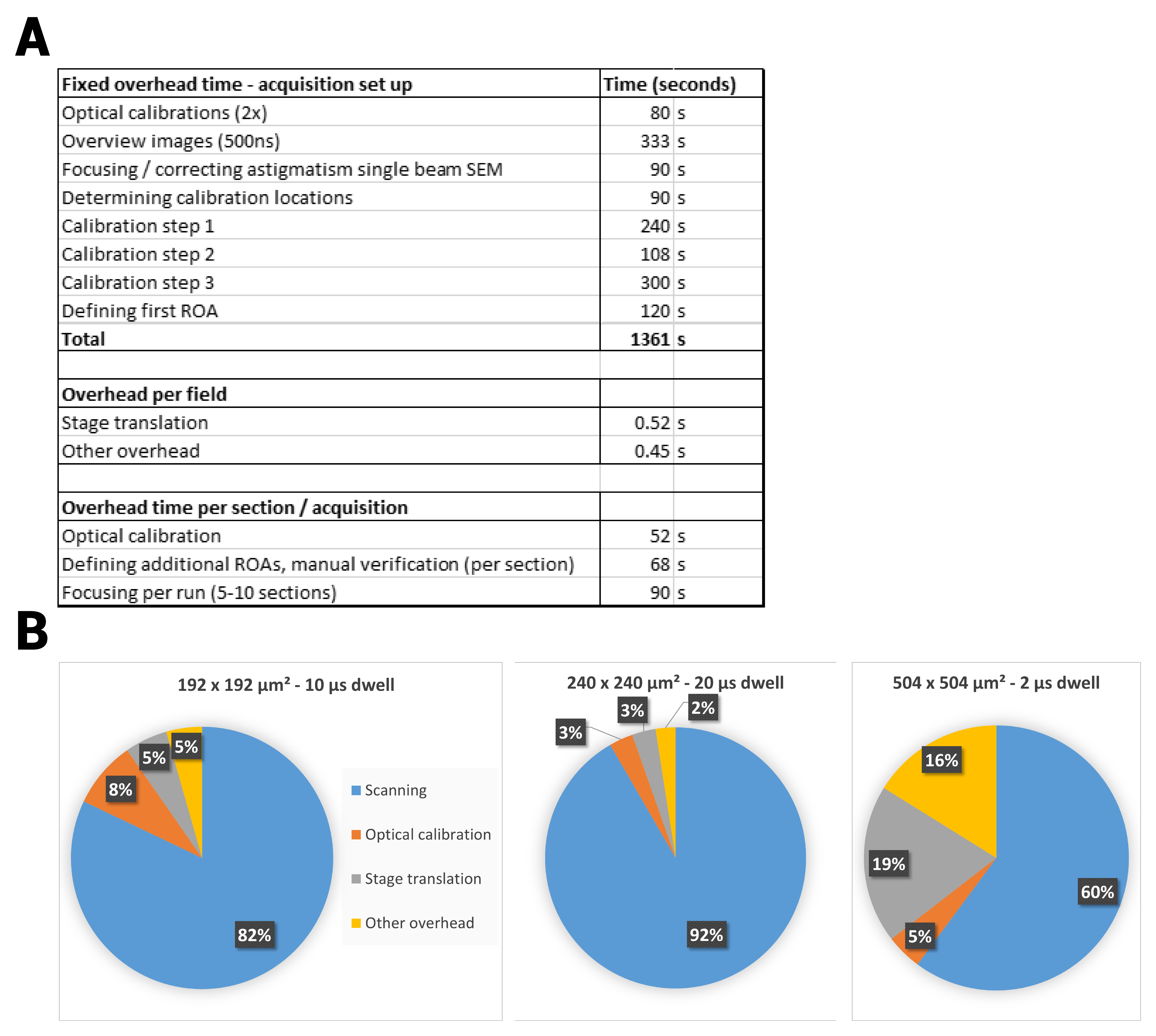

Supplement: Supplementary file 1 — Supplementary Material Details [file j_mim-2024-0005_suppl_001.zip › Supplementary_figures/S6.png]
