# Supplementary material for: FAST-EM array tomography: a workflow for multibeam volume electron microscopy
Source: Methods Microsc. 2024 Jul 11;1(1):49–64. doi: 10.1515/mim-2024-0005 (PMC11308914; doi:10.1515/mim-2024-0005)
Supplement: Supplementary file 2 — Supplementary Material Details [file j_mim-2024-0005_suppl_002.docx]

Appendix

Figure S1: ODEMIS FAST-EM graphical user interface, overview tab. 1: Scintillator selection and ROA definition tools. 2. Single-beam tool. 3. Optical calibration, detector (single-beam) settings, overview image settings.

Figure S2: ODEMIS FAST-EM graphical user interface, acquisition tab. 4. Calibration step settings. 5. Project and ROA settings.

Figure S3: vEM reconstruction of an islet of Langerhans in rat pancreas tissue. **A**: Overview images of sections, showing a zoom in on a single ROA, a single field and a single cell respectively. **B**: Aligned volume reconstruction from 44 100nm serial sections showing the orthogonal reslices through the center of the stack (xz and yz). **C**: Volume rendering of the full (continuous) stack. Inset shows smaller subvolume at 8nm/pixel resolution with arrows pointing at structures of interest (Star indicators: *=Mitochondrium, **=Lysosome, ***=Nuclear membrane). The data quality and alignment is consistent throughout the stack.

Figure S4: FAST-EM resolves organelle structures. **A**: xy, xz and yz planes of data in Fig. 5C at 8nm/pixel resolution. The axial resolution (100nm) and fine alignment allow identification of organelles in xz and yz planes. **B**: High resolution crops of MCF-7 cells stained with uranyl acetate (see Table 1 for details). Several organelles are indicated with arrows. FAST-EM resolves the detailed structure of several organelles, including mitochondria cristae, stacked golgi membranes, double membranes.

Figure S5: Automatic instance segmentation of mitochondria in FAST-EM data of rat pancreas using MitoNet. **A**: MitoNet predictions on subset of data, showing the orthogonal slices at the locations indicated by the red cross, and 3D renderings in Napari. **B**: Ground truth annotations of mitochondria from the same volume. The predictions show qualitative reasonable overlap with the ground truth.

Figure S6: A: Set up, per-field and per-section overhead times for FAST-EM. B: Time spent on scanning and overhead, relatively, for acquisitions in this paper and a large volume. For the dwell times considered here, the acquisition time is mostly limited by the scan speed.

Table S1:  Estimated reconstruction times for FAST-EM of a 500 x 500 x 50 μm^3^ volume from 500 serial sections.

| **Post-processing step** | **Compute time** | **Unit** |
| --- | --- | --- |
| Post-correction | 90.05 | h |
| Import | 50.88 | h |
| Stitching | 139.60 | h |
| Rough alignment | 0.19 | h |
| Fine alignment | 734.72 | h |
| Export | 300.18 | h |
| Total | 1315.63 | h |
|  | 54.82 | days |
